# Supplementary material for: Identification of African Swine Fever Virus Inhibitors through High Performance Virtual Screening Using Machine Learning
Source: Int J Mol Sci. 2021 Dec 14;22(24):13414. doi: 10.3390/ijms222413414 (PMC8703626; doi:10.3390/ijms222413414)
Supplement: Supplementary file 1 [file ijms-22-13414-s001.zip › ijms-1447808-supplementary.pdf]

Supplementary Materials

Table S1. The dataset of selected crystal structures of ASFV protein used in this study

PDB ID	Sequence length	Organism	Resolution(Å)	Mutations	Released
5HRD	178	African swine fever virus	1.8	No	2017-01-28
5HRG	178	African swine fever virus	2.0	Yes [D51N]	2017-01-28
5HRL	178	African swine fever virus	2.4	No	2017-01-28
5HRI	178	African swine fever virus	2.2	No	2017-01-28
5HR9	178	African swine fever virus	2.2	Yes [L52/163M]	2017-01-28
5HRK	178	African swine fever virus	2.9	Yes [H115F]	2017-01-28
5HRE	178	African swine fever virus	1.75	No	2017-01-28
5HRB	178	African swine fever virus	1.70	No	2017-01-28
5HRH	178	African swine fever virus	3.0	Yes [H115F/R127A]	2017-01-28
5HRF	178	African swine fever virus	2.25	No	2017-01-28
5XM9	177	African swine fever virus BA71V	3.05	Yes [D51N]	2018-01-24
5XMA	177	African swine fever virus BA71V	3.80	No	2018-01-24
5XM8	177	African swine fever virus BA71V	2.55	No	2018-01-24

Table S2. The SiteMap parameters of the selected druggable pockets used in this study.

PDB ID	Dscore	SiteScore	Size	Volume	Exposure	Enclosure	Contact	Phobic	Philic	Balance	Don-Acc
5HRL	0.802	0.953	79	254.849	0.518	0.75	0.95	0.434	1.483	0.293	0.784
5HRI	0.762	0.853	69	255.192	0.603	0.664	0.803	0.36	1.28	0.281	0.463
5HR9	0.726	0.855	70	189.336	0.583	0.66	0.828	0.137	1.401	0.098	0.742
5HRF	0.705	0.763	59	146.804	0.74	0.597	0.692	0.143	1.151	0.124	1.355
5HRD	0.695	0.809	48	162.582	0.505	0.75	0.937	0.621	1.246	0.499	2.033
5XM9	0.64	0.684	42	127.596	0.757	0.604	0.681	0.193	1.017	0.19	1.018
5HRE	0.608	0.744	35	166.698	0.615	0.763	0.946	0.339	1.245	0.272	0.754
5HRB	0.601	0.719	44	133.427	0.659	0.647	0.803	0.22	1.261	0.174	1.22
5HRG	0.598	0.81	40	180.075	0.403	0.817	1.105	0.452	1.494	0.303	0.967
5XMA	0.583	0.68	39	129.654	0.727	0.631	0.7	0.122	1.173	0.104	1.392
5HRH	0.543	0.693	38	147.147	0.658	0.659	0.805	0.089	1.324	0.067	0.497
5XM8	0.536	0.646	34	124.852	0.779	0.626	0.681	0.082	1.189	0.069	1.476
5HRK	0.479	0.727	42	127.253	0.625	0.676	0.87	0	1.643	0	0.725

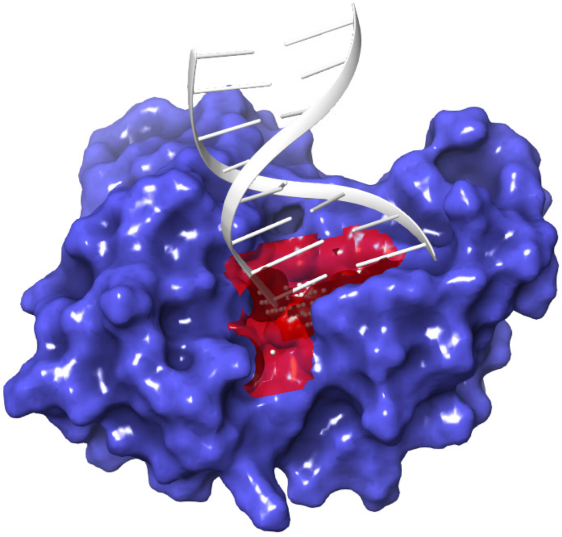

Figure S1. The potential binding pockets identified by SiteMap on the DNA complex structures of *Asfv*PolX. Colored spaces represent SiteMap predictions for the DNA binding surface pockets on the *Asfv*PolX protein (PDB ID: 5HRG, in red surface representation). Gray dots represent the cavity of the putative active site of the protein. *Asfv*PolX protein and DNA are shown as surface and cartoon, respectively.

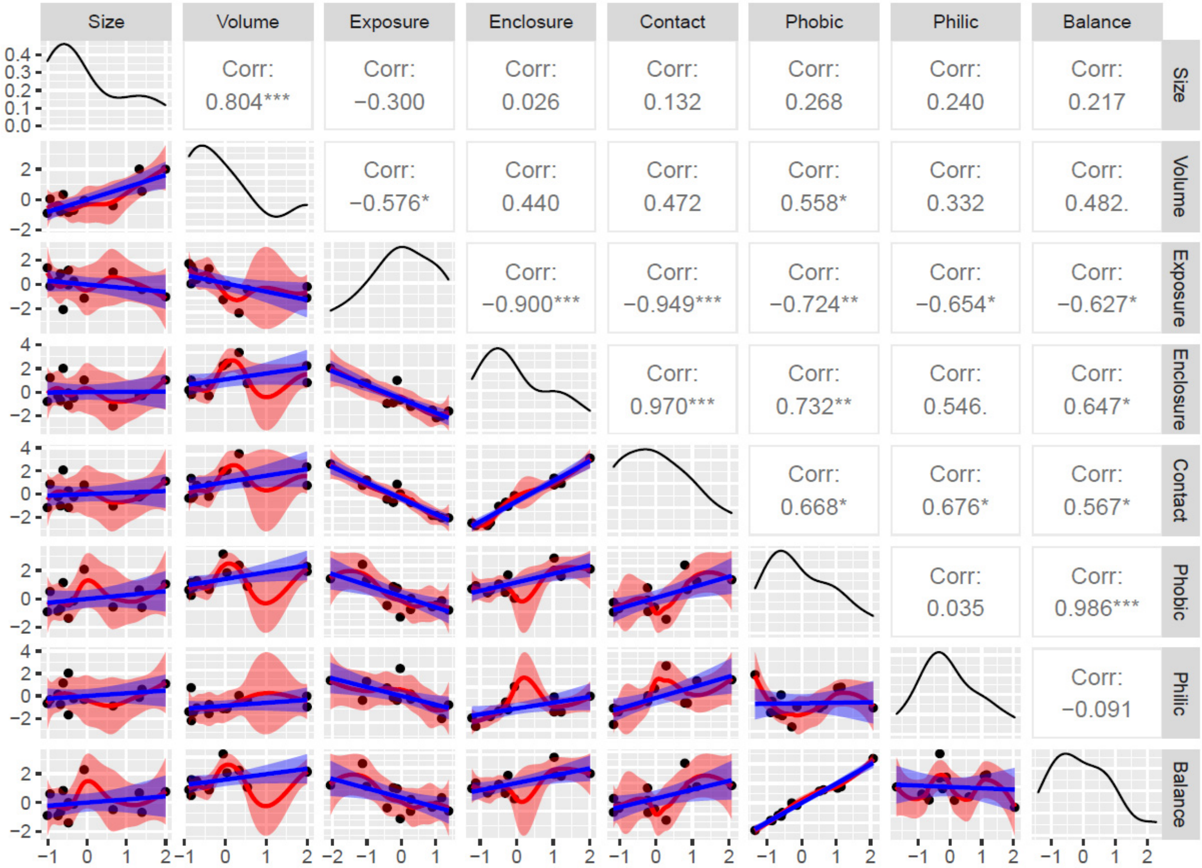

Figure S2. Pearson correlation matrix for binding site parameters identified using the SiteMap module

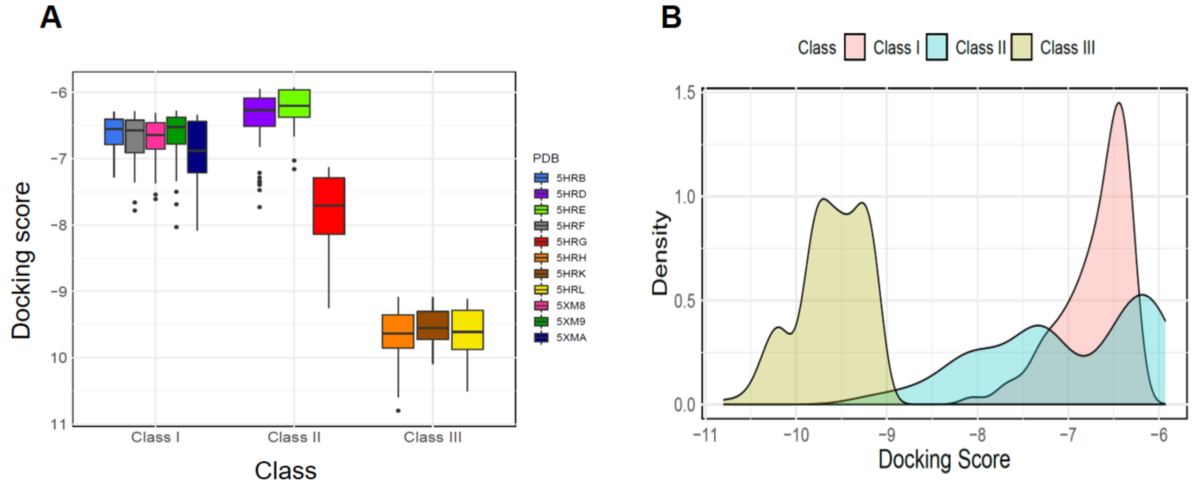

Figure S3. Comparison of the docking performance of three classes across the *Asfv*PolX dataset. (A) Boxplots for Glide docking score distributions of each crystal structure. The black line indicates the median result for each crystal structures. (B) The probability density curves of the Glide docking score. The X-axis represents the docking score of each structure-ligand pair, and the Y-axis represents the probability density

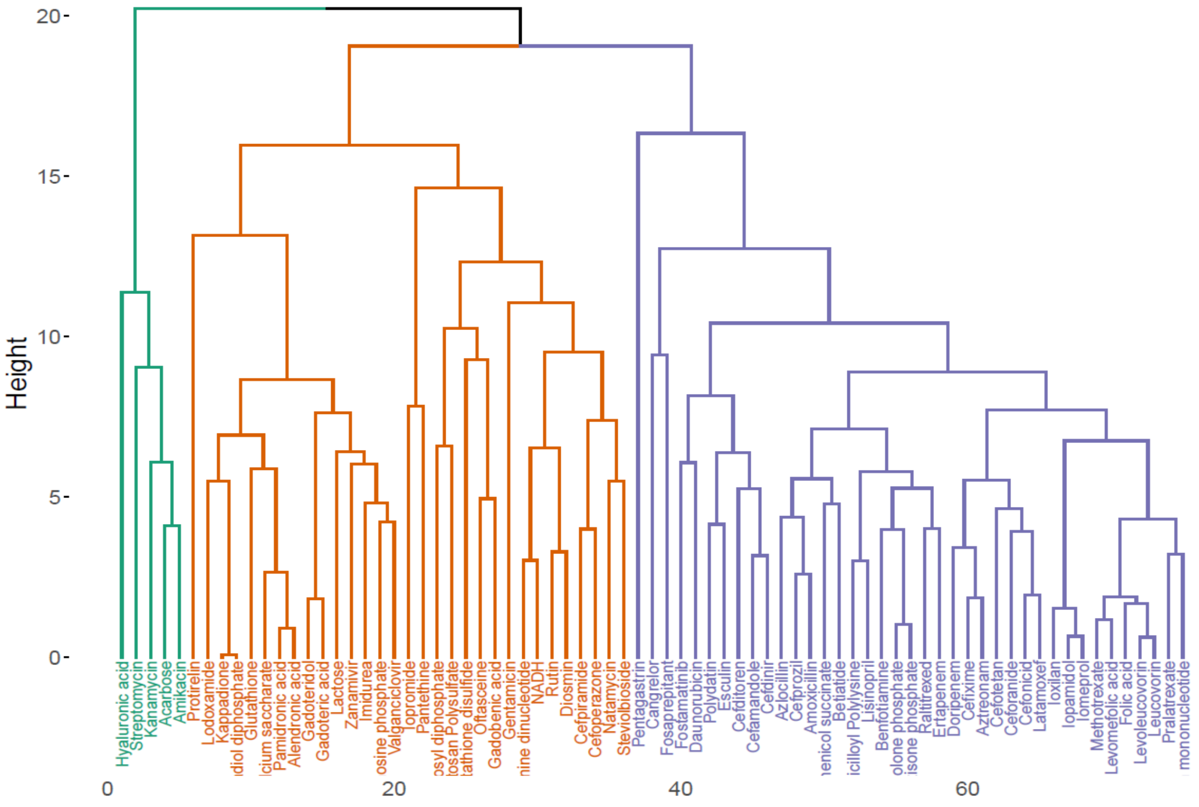

Figure S4. Circular HCA dendrogram of cluster 2 using Euclidean distances and average linkage showing three clusters. Each cluster is colored differently.

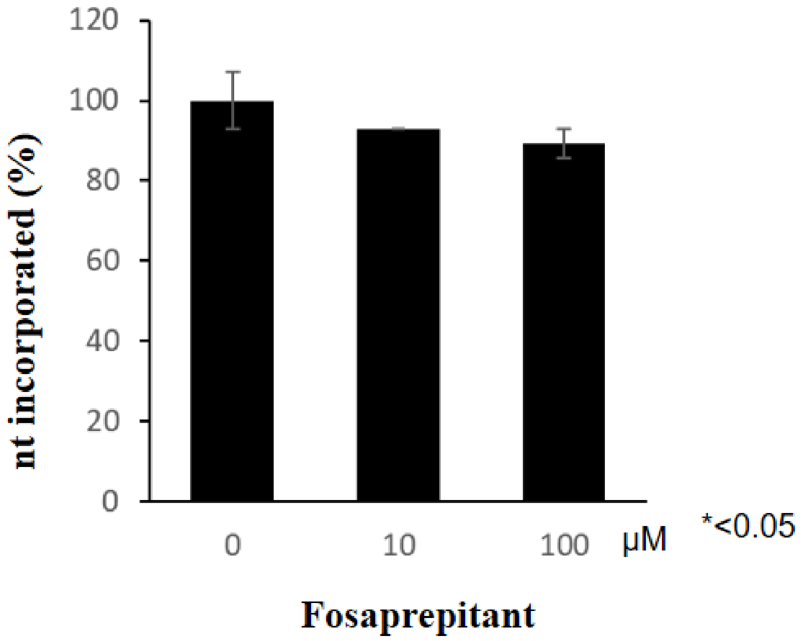

Figure S5. Fosaprepitant was mixed with 100 ng of ASFV PolX for 10 min and polymerase activities were measured as described in Materials and Methods.
